# Psychiatric disorder as clinical presentation of primary Sjögren's syndrome: two case reports

**DOI:** 10.1186/1744-859X-9-12

**Published:** 2010-04-01

**Authors:** Lorenzo Pelizza, Federica Bonacini, Alberto Ferrari

**Affiliations:** 1 Guastalla Psychiatric Service, Reggio Emilia Mental Health Department, Reggio Emilia, Italy

## Abstract

Psychiatric disorders in primary Sjögren's syndrome constitute a possible clinical reality that each practitioner must be able to recognise and treat. In this article, two case reports of mental disorder as clinical presentation of primary Sjögren's syndrome are presented, suggesting that psychiatric manifestations in primary Sjögren's syndrome can occur not only during its longitudinal course, but also at the onset of the autoimmune syndrome. A better adapted prescription of corticosteroids and/or immunosuppressive agents (together with specific psychotropic treatments) can induce rapid relief of mental symptoms.

## Background

Sjögren's syndrome (SS) is considered to be one of the most common autoimmune diseases with a prevalence thought to equal or exceed that of rheumatoid arthritis (2% to 4% of the population) [1]. It is characterised by a chronic inflammation of the lachrymal and salivary glands, which leads to exocrine hypofunction and the well recognised clinical features of dry eyes (xerophthalmia) and dry mouth (xerostomia). Other exocrine glands may also be affected, causing a wider variety of complaints (for example, cough, pancreatic insufficiency, chronic sinusitis, gingivitis and laryngitis).

In isolation, SS is termed 'primary' and when in combination with another autoimmune disease (such as systemic lupus erythematosus (SLE) or rheumatoid arthritis (RA)), it is termed 'secondary'. The distinction between secondary and primary SS is difficult and a source of debate in the rheumatological literature. However, there seems to be a moderate specificity of different types of anti-nuclear antibodies (ANA) for different rheumatological illnesses. In 50% to 80% of patients with SS, anti-Ro/SS antigen A (SS-A) and/or anti-La/SS antigen B (SS-B) antibodies (directed against a small intracellular RNA-protein complex) are detected [2].

Studies of SS have used a variety of classification criteria, leading to difficulties when trying to compare results. In response to this, a European Community (EC) criteria set was developed to standardise the definition of SS for use in research study [3] (see Appendix 1).

## Primary SS and psychiatric disorders

Some investigators have recently documented higher rates of central nervous system involvement in primary SS (CNS-SS) than previously noted [4]. These CNS complications include focal neurological deficits, diffuse cerebral involvement (that is, aseptic meningitis, vascular encephalopathy, dementia), and psychiatric disorders. Although primary SS generally does not affect the brain, most clinicians often fail to consider CNS-SS manifestations in their differential diagnosis [1].

Cox and Hales [5] have suggested that the incidence of mild to moderate psychiatric and/or cognitive impairment may be as high as 80% in patients with CNS-SS. Mental disorder seems to occur most commonly as an 'atypical' mood disorder (characterised by a combination of depression, agitation, irritability, somatic symptoms (that is, headache, gastrointestinal disturbances), and mild memory impairment with attention and concentration deficits), which leads to a higher degree of distress and a lower sense of wellbeing. According to Stevenson *et al. *[6], subjects with primary SS are at increased risk of clinical depression. Therefore, an early recognition and an appropriate intervention are essential to reduce the negative impact of depression on quality of life and outcome.

Moreover, schizophrenia-like psychosis, along with symptoms such as somatisation, anxiety, panic attacks, obsessive-compulsive disorder, and abnormal personality traits (such as hostility, paranoid ideation, hypochondriac and histrionic features), have also been described [5]. Those findings strongly suggest that mental disorders are common in primary SS patients, who then may need psychiatric help and appropriate psychotropic therapy.

The neuropathological mechanisms of SS are unknown. In CNS-SS, the most serious neuropsychiatric disorder appears to result from ischaemic damage caused by vasculitis [7], but the role of anti-Ro/SS-A alone or in combination with anti-La/SS-B is still unclear. Ampelas *et al. *[8] have also suggested that mental disorders in primary SS were explained by secondary psychological distress. According to them, the slowly progressive and fluctuating course of SS created constant discomfort from ocular and oral dryness, dysphagia, dyspareunia and functional disability, inducing a depressive or anxious reaction to a chronic illness. They reported that drugs with anticholinergic side effects (such as tricyclic antidepressants), which enhanced oral dryness, had to be avoided, and that social and psychological support was also important.

In the present article, we report two cases of mental disorders as clinical presentations of primary SS, suggesting that psychiatrists should keep in mind that SS can be a potential cause of psychiatric manifestations when examining patients with multiple unexplained somatic complaints and psychopathological symptoms (especially if 'atypical' or 'treatment refractory'). Moreover, we want to emphasise that psychiatric disorders in primary SS can occur not only during its longitudinal course, but also at the onset of SS.

## Case presentations

### Case report 1

The patient was a 42-year-old woman who came to our outpatient psychiatry service for the treatment of a depressive episode, characterised by depressed mood, anhedonia, anorexia with weight loss, hypersomnia, diminished libido, and poor concentration. She also reported hopelessness, feelings of sadness, and difficulty at work due to persistent fatigue and abulia. At 2 months prior to her admission, the patient had responded to sertraline at a dose of 75 mg/day, but she stopped the medication because of side effects (sedation and nausea). Her depressive symptoms then recurred and her work difficulty continued.

According to the Diagnostic and Statistical Manual of Mental Disorders, fourth edition, text revision (DSM-IV-TR) criteria [9], a 'major depressive disorder - single episode' was diagnosed. Bupropion (at a dose of 150 mg/day) was given and partially relieved depressive symptoms (particularly depressed mood, hopelessness, and anhedonia), but did not adequately address fatigue and abulia. Bupropion was administered in order to its smaller propensity for causing sedation and gastrointestinal side effects than selective serotonin reuptake inhibitors (SSRIs).

For a better comprehension of fatigue and abulia, biochemical analysis was conducted. Laboratory findings of inflammatory processes (erythrocyte sedimentation rate elevation (90 mm in the first hour), mild normochromic and normocytic anaemia (haemoglobin serum concentration of 10.3 g/dl), and polyclonal hypergammaglobulinaemia (IgG serum level of 2174 mg/dl)) required a review of the patient's medical history, which showed sinus problems and an episode of conjunctivitis in the last 3 weeks. Focused questioning also revealed recent subjective complaints of myalgia, dry eyes (sandy feeling) and dry mouth. The history of these somatic manifestations suggested their later onset than the depressive symptoms (which had begun at least 2 months earlier).

A Schirmer test (to quantify tear production) was positive (< 5 mm in 5 min) and a gingival biopsy showed a focal lymphocytic sialoadenitis (with a focus score of 2 per mm^2 ^of glandular tissue) without mucosal lesion. The patient's ANA titre was positive (at a dilution of 1:80), as well as anti-Ro/SS-A antibody test (at a dilution of 1:320). Anti-La/SS-B titre, other SLE and RA markers, and tests of the complement system (C_3 _and C_4_) were negative. Electroencephalogram (EEG) and magnetic resonance imaging (MRI) showed no biological abnormality.

According to the EC criteria for the classification of SS (Appendix 1), a diagnosis of 'primary SS' was formulated. Prednisone at 5 mg/day was given. After being treated with bupropion (300 mg/day) and prednisone (10 mg/day) for 1 month, her fatigue, myalgia, dry eyes and dry mouth improved, and her residual depressive symptoms improved further. She is currently being maintained on prednisone at a dose of 7.5 mg/day. Her only psychiatric medication is bupropion at a dose of 150 mg/day.

### Case report 2

The patient was a 35-year-old woman who was admitted to our psychiatric hospital for agitation in August 2008. In the 2 months prior to hospitalisation she showed at least three unexpected anxiety episodes with panic features, characterised by palpitations, chest pain, hot flashes, sweating, nausea, trembling and fear of dying. Each episode had been followed by persistent concern about having additional panic attacks and agoraphobia. Phobic situations (such as being outside the home alone and travelling in a bus or in a car) were avoided. The panic attacks were not due to the direct physiological effects of a psychoactive substance or a general medical condition, and were not better accounted for by another mental disorder. The patient had no history of psychiatric and/or somatic illness. At the admission, she referred to a stable and previously satisfying married life and work, and complained of agitation, irritability, difficulty in sleeping, poor concentration, increasing fatigue, weakness, and demoralisation (related to persistent concern about having additional panic attacks). EEG, MRI and a neurological examination were normal.

According to the DSM-IV-TR criteria [9], a diagnosis of 'panic disorder with agoraphobia' was formulated. On DSM-IV-TR axis II, a 'histrionic personality disorder' was diagnosed (structured clinical interview for DSM-IV axis II personality disorders (SCID-II)). Paroxetine (at a dose of 20 mg/day) and alprazolam (at a dose of 2 mg/day) were given and partially relieved the anxiety symptoms, but did not adequately address fatigue, weakness, and demoralisation. She was discharged to a convalescent home with the prescription to continue her therapy.

In October 2008, the patient was readmitted for agitation. She had stopped medications because of side effects (sedation) and her anxious-depressive symptoms recurred. At her initial medical evaluation, she complained of persistent fatigue, myalgia, and symptoms referred to her eyes (sandy feeling, itchiness, and burning sensation), mouth (dryness), and joints (pain and stiffness, mostly in the elbows and ankles). Laboratory findings revealed an erythrocyte sedimentation rate elevation (95 mm in the first hour) and a mild lymphopoenia of 700 cells per ml. Rheumatoid factor was undetectable and tests of the complement system (C_3 _and C_4_) were negative. The patient's ANA titre was positive (at a dilution of 1:80), as well as anti-Ro/SS-A antibody test (at a dilution of 1:120). Anti-La/SS-B titre and other SLE markers were negative. A Schirmer test was positive (< 5 mm in 5 min) and salivary gland scintigraphy showed a poor fixation of the radionuclide. Additionally, a stimulation test revealed no further functional activity of the salivary glands. Those findings were characteristic of the sialoadenitis typically observed in primary SS [1]. Salivary gland biopsy was not conducted.

According to the EC criteria for the classification of SS (Appendix 1), a diagnosis of 'primary SS' was made. Prednisolone was given at a dose of 40 mg/day and was tapered gradually using alternate day method. After being treated with prednisolone, citalopram (at a dose of 40 mg/day), and lorazepam (at a dose of 5 mg/day) for 2 months, her fatigue, myalgia, arthralgia, dry eyes and mouth improved, and her anxious-depressive symptoms gradually disappeared. She is currently being maintained on prednisolone at a dose of 20 mg/day. Her only psychiatric medication is citalopram at a dose of 30 mg/day.

## Conclusions

In the present article, we report two cases of primary SS with a psychiatric disorder as clinical presentation (major depression and panic disorder with agoraphobia), suggesting that a mental disorder can be not only a CNS-SS complication, but also an early manifestation of SS, which precedes the well recognised glandular 'key symptoms' of the autoimmune syndrome (xerophthalmia and xerostomia). This evidence leads to important clinical considerations.

Psychiatric disorders in primary SS constitute a possible clinical reality that each psychiatrist must be able to recognise and treat. Therefore, when examining patients with multiple somatic complaints and mental symptoms, psychiatrist should search for SS autoantibodies in the serum (after a careful physical examination to emphasise signs of dry mouth and dry eyes), because the diagnosis of primary SS could lead to a better adapted prescription of corticosteroids and/or immunosuppressive agents, which (together with specific psychotropic drugs) can induce remarkably rapid relief from the psychiatric disorder. In general, this consideration holds for all autoimmune diseases.

Psychiatric presentation of primary SS also suggests that mental disorders do not occur only as a response to a psychological distress or a reaction to a chronic rheumatological disease, but may be an early manifestation of the same autoimmune process, which presumes the direct immunological activity of SS on the central nervous system (by T cells, autoantibodies, cytokines, or the programmed cell death (apoptosis)) [10].

Finally, rheumatologists and other clinicians (that is, neurologists, ophthalmologists, dentists) should keep in mind that mental disorders are possible in primary SS patients (both at the onset and during the longitudinal course of the autoimmune syndrome) and then may need psychiatric help and additional appropriate psychotropic therapy, which can lead to remarkably rapid relief of mental symptoms.

## Competing interests

The authors declare that they have no competing interests.

## Authors' contributions

LP and FB conceived the study and participated in its design. AF participated in coordination and helped to draft the manuscript. All authors read and approved the final manuscript.

## Consent

Written informed consent was obtained from the patients for publication of those case reports and any accompanying images. A copy of the written consent is available for review by the Editor-in-Chief of this journal.

## Appendix 1

European Community (EC) criteria for the classification of Sjögren's syndrome (SS) [3].

**1. Ocular symptoms**. A positive response to at least one of the following questions:

(a) Have you had daily, persistent, troublesome dry eyes for more than 3 months?

(b) Do you have a recurrent sensation of sand or gravel in the eyes?

(c) Do you use tear substitutes more than three times a day?

**2. Oral symptoms**. A positive response to at least one of the following questions:

(a) Have you had a daily feeling of dry mouth for more than 3 months?

(b) Have you had recurrently or persistently swollen salivary glands as an adult?

(c) Do you frequently drink liquids to aid in swallowing dry food?

**3. Ocular signs**. Objective evidence of ocular involvement defined as a positive result for at least one of the following tests:

(a) Schirmer's test, performed without anaesthesia (≤ 5 mm in 5 min).

(b) Rose bengal score or other ocular dye score (≥ 4 according to Van Bijsterveld's scoring system).

**4. Histopathology**. In minor salivary glands (obtained through normal-appearing mucosa), evidence of focal lymphocytic sialoadenitis, evaluated by an expert histopathologist, with a focus score ≥ 1 (defined as a number of lymphocytic foci (adjacent to normal-appearing mucous acini and contain more than 50 lymphocytes) per 4 mm^2 ^of glandular tissue).

**5. Salivary gland involvement**. Objective evidence of salivary gland involvement, defined by a positive result for at least one of the following diagnostic tests:

(a) Unstimulated whole salivary flow (≤ 1.5 ml in 15 min).

(b) Parotid sialography showing the presence of diffuse sialectasias (with punctate, cavitary, or destructive pattern) without evidence of obstruction in the major ducts.

(c) Salivary scintigraphy showing delayed uptake, reduced concentration and/or delayed excretion of tracer.

**6. Autoantibodies**. Presence in the serum of the following autoantibodies: antibodies to Ro (SS-A) or La (SS-B) antigens, or both.

## Revised rules for classification

**1. For primary SS**. In patients without any potentially associated disease, primary SS may be defined as follows:

(a) The presence of any four of the six items is indicative of primary SS, as long as either item 4 (Histopathology) or 6 (Serology) is positive.

(b) The presence of any three of the four objective criteria items (items 3, 4, 5, 6).

(c) The classification tree procedure represents a valid alternative method for classification, although it should be more properly used in clinical-epidemiological survey.

**2. For secondary SS**. In patients with a potentially associated disease (for instance, another autoimmune disorder), the presence of item 1 or item 2 plus any two from among items 3, 4, and 5 may be considered as indicative of secondary SS.

## Exclusive criteria

Past head and neck radiation therapy, hepatitis C infection, AIDS, pre-existing lymphoma, sarcoidosis, graft versus host disease, use of anticholinergic drugs (for a time shorter than fourfold life of the drug).
